# Assessment of Occupational Burnout among Intensive Care Unit Staff in Jazan, Saudi Arabia, Using the Maslach Burnout Inventory

**DOI:** 10.1155/2022/1298887

**Published:** 2022-04-16

**Authors:** Abdullah Shbeer, Mohammed Ageel

**Affiliations:** College of Medicine, Jazan University, Jazan, Saudi Arabia

## Abstract

**Objective:**

ICU workers are among the healthcare staff exposed to high occupational burnout in their daily interactions with patients, especially during the COVID-19 pandemic. This study aimed to investigate the prevalence and risk factors of burnout among ICU staff in the Jazan region of Saudi Arabia.

**Methods:**

A cross-sectional study was conducted using the Maslach Burnout Inventory (MBI), which was distributed to ICU staff between August 1 and November 30, 2021. A total of 150 ICU workers were invited to participate in the study.

**Results:**

A total of 104 ICU staff responded to the survey (69% response rate), including 62 nurses, 30 physicians, and 12 respiratory therapists. Among the respondents, 63 (61%) were female and 41 (39%) were male. The mean scores for emotional exhaustion, depersonalization, and personal accomplishment were 22.44 ± 14.92, 9.18 ± 7.44, and 29.58 ± 12.53, respectively. The ICU staff at high risk of emotional exhaustion, depersonalization, and personal accomplishment were 36%, 28%, and 47%, respectively. The leading cause of burnout among ICU staff in the study was workload, and taking a vacation was the most cited coping mechanism for occupational burnout.

**Conclusion:**

ICU staff are at high risk of emotional exhaustion, depersonalization, and lack of personal accomplishment. Policymakers should implement regulations that ensure hospitals have adequate employees to reduce the workload that leads to occupational burnout.

## 1. Introduction

Healthcare is a complex field, and its professions are some of the most demanding because practitioners' lives are put at risk. However, the staff, including qualified registered nurses, medical equipment technicians, physicians, and respiratory technicians working in intensive care units (ICU), face even more challenging situations compared to typical nursing professions [1]. According to [2], high expectations on performance and understanding can exert enormous pressure on intensive care personnel. In Saudi Arabia, in particular, there is a scarcity of highly qualified staff compared to the usual influx of patients admitted to ICU hospitals [3]. The disproportionate ratio of a high number of ICU patients to limited staff is contributing to severe psychological stress and deteriorating mental health [4]. Anxiety, depression, sleep disorders, and burnout syndrome are related to occupational psychosocial risk factors in healthcare sector in terms of work-related stress (high workload, poor organization, and rewards), workplace violence, and high emotional loads [5, 6]. Burnout is defined as a syndrome characterized by high emotional exhaustion, cynicism, and low personal accomplishment [7]. Burnout has a high prevalence in healthcare individuals, before and after the pandemic, leading to absenteeism, high turnover and leave intention, suicide, and posttraumatic stress disorders [8]. To accurately assess the occupational burnout experienced by ICU workers, the Maslach Burnout Inventory (MBI) is a viable tool [9].

The psychosomatic factors and burnout subscales derived from MBI are some of the indicators of ICU-bound staff working under highly stressful conditions [10]. Furthermore, the onslaught of stress for long periods can cause ICU staff to become sleep deprived and devoid of motivation, which eventually impairs their cognition and decision-making skills, exposing critical patients to errors [11]. According to [12], their analysis showed severe level prevalence, i.e., 65.9% of burnout among critical care professionals at King Saud Medical City (KSMC) in Saudi Arabia-Riyadh. As is apparent, the calculated risks are real and not just theoretical. In addition, multiple surveys were conducted using MBI in multiple hospitals in Saudi Arabia, which revealed that ICU staff are heading toward a total mental breakdown on a large scale. According to [13], the prevalence of burnout syndrome (BOS) in Saudi Arabia will continue to rise exponentially unless an intervention is introduced. It has been proposed that hospitals in Saudi Arabia should increase the salaries and bonuses for their ICU staff, so that a higher number of qualified ICU nurses, physicians, and respiratory technicians show interest in working in Saudi Arabia [3]. Furthermore, it has been acknowledged that Saudi Arabian hospitals do not have enough staff; however, if more professionals are recruited, it will be possible to provide them relaxation by giving them shorter shifts, fewer patients to look after, and more slots of employees changing than usual [14]. Additionally, ICU services are already a healthcare field in which employees are already in great demand, so their job satisfaction should be protected and burnout minimized [13]. As argued by [15], more than 65% of ICU staff have reported that they feel emotionally exhausted, and this number is expected to be much higher if every ICU specialist is completely honest. Furthermore, only 27% reported that they were satisfied with their income, considering the work that they do [16]. According to [17], the adoption of evidence-based practice in Saudi Arabia would optimize the work of ICU staff, reducing their mental and psychological strain.

While ICU staff possess an inherent capability to persevere under stressful conditions, there eventually comes a time when their mental guard is weakened and withdrawn [18]. However, to assess the burnout scenario more accurately, a custom-tailored MBI, along with an adequate questionnaire, needs to be administered [3]. ICU professionals, including registered nurses, physicians, and respiratory technicians, play an indispensable role in protecting lives. Accordingly, the Saudi Arabian government even encourages governmental interventions for managing work-related stress [19]. According to [20], the human error caused by the inability to think and make critical decisions among ICU staff will become a major crisis in the near future. Hospital administration should be keen enough to ensure that ICU staff are free of burnout. According to [21], ICU clinicians should be regularly tested for burnout to ensure their well-being. Therefore, this study aimed to investigate the prevalence and risk factors of burnout among ICU staff in the Jazan region of Saudi Arabia.

## 2. Materials and Methods

A cross-sectional study was conducted in the Jazan region of Saudi Arabia, between August 1 and November 30, 2021, to assess the extent of occupational burnout among ICU staff in private and public hospitals. A total of 150 ICU employees from five major hospitals were invited to participate in the study based on the contact information provided by the hospitals for full-time ICU staff. The ICU staff included in this study were physicians, respiratory therapists, and nurses. This study was approved by the Ethical Committee at Jazan University. The questionnaire and participation consent form were e-mailed to the identified ICU employees via a link. The initial email was followed by two monthly reminders to increase the response rate. A total of 104 participants responded and filled out the survey (about 70% response rate), which produced fairly accurate results after considering medians and variances. A response rate of greater than 60% may be considered sufficient for most research purposes [22].

The survey was based on the MBI tool, which was deemed suitable for the chosen subjects [23]. The MBI tool is considered a competitively feasible tool for the current requirement because it is one of the most validated and well-tested tools for calculating employee burnout available in the medical industry today [24]. However, to use this tool, a license is required from an affiliated institutional body. In this case, the Mind Garden Company was consulted for licensing and permission. The MBI tool comprises 22 questions specifically designed to address the precision of the study. The MBI tool used in this study was the English version. To specifically incorporate the coping techniques, two open-ended questions were added to the set of questions. The MBI tool is used to determine the three distinct phases of BOS, primarily in terms of lack of personal accomplishment (eight questions), depersonalization (five questions), and emotional exhaustion (nine questions). Each question holds a gradual scoring weight, according to which participants are finally categorized as high, moderate, and low level of burnout [24]. The MBI questions were constructed to connect with the personal feelings and cognition of the medical professionals picked from the ICU. The answers are designed to vary between the two extremities. A full score of seven points indicates a complete likelihood, while a zero score indicates a zero possibility of occurrence. Finally, the scores gathered were superimposed to generate a mean score that would indicate the probability and overall extent of depersonalization and emotional exhaustion describing burnout. Conversely, regarding personal accomplishment, a high value for burnout translates into the possibility of lower mean scores.

For interpretation of MBI results, the normative data, means, and standard deviations (SD), for the MBI subscales that have been reported from an overall USA population sample was used [25]. The following benchmarks (reference levels defined for healthcare workers) were considered to assess the level of occupational burnout [26]:

- Emotional exhaustion: low (≤16 pts), medium (17–27 pts), high (≥28 pts);
- Depersonalization: low (≤5 pts), medium (6–10 pts), high (≥11 pts);
- Personal accomplishment: low (≥37 pts), medium (36–30 pts), high (≤29 pts).

Alongside the MBI tool, sociodemographic questions and two open-ended questions were asked about coping mechanisms and factors identified by ICU staff that cause occupational burnout.

The collected data were entered into MS Excel and transferred to SPSS (Statistical Package for Social Sciences, Version 25, SPSS, Inc., Armonk, NY) for statistical analysis. Descriptive analyses were performed to obtain the mean and standard deviation (SD) for the MBI subscale scores. The burnout levels for the ICU staff were obtained. An independent sample *t*-test and one-way analysis of variance (ANOVA) were used to assess the statistical differences between variables. A *p* value < 0.05 was used to indicate statistical significance.

## 3. Results

A total of 104 ICU staff responded to the survey, including 62 nurses, 30 physicians, and 12 respiratory therapists (61% female and 39% male).  Table 1 provides the sociodemographic variables and the environmental and work variables. Most participants were in the age group of 25–34 years (58%). Most had a bachelor's degree (69%), 0–5 years of work experience (38%), and work in public hospitals (79%). About 70% of the ICU staff reported working more than six overtime hours weekly and 73% had alternate shift duties. Most subjects (87%) reported a good perception of their health status and 62% reported job satisfaction. Most of the participants received monthly salaries between 5000 and 10000 SAR.

As given in Table 2, the mean emotional exhaustion score for the ICU staff was 22.44, which is higher than the MBI norm sample (21.0). Similarly, the mean depersonalization score (9.18) for the ICU staff was higher than the mean score for the MBI norm sample (8.7). The ICU staff's mean score for personal accomplishment means was 29.58, which is lower than the MBI norm sample (34.6). A lower mean score for personal accomplishment indicates a higher level of burnout. Overall, the level of occupational burnout among ICU staff was higher for all subscales compared to the MBI norm sample.

Table 3 provides the MBI subscale scores for ICU staff. Occupational burnout is categorized into three levels: high, medium, and low. In emotional exhaustion, the high level of burnout was indicated in 34% of the ICU staff and was the highest among respiratory therapists (50%), followed by nurses at 37% and, finally, physicians at 23%. The high level of depersonalization accounted for 27% of the ICU staff, with the highest occurrence among nurses (43.5%), followed by respiratory therapists (33.3%) and physicians (24%). Regarding personal accomplishment, the high level was indicated among 45% of the ICU staff. Physicians were the highest group at risk with 53%, followed by nurses at 27% and 6% among respiratory therapists.

**Figure 1:**
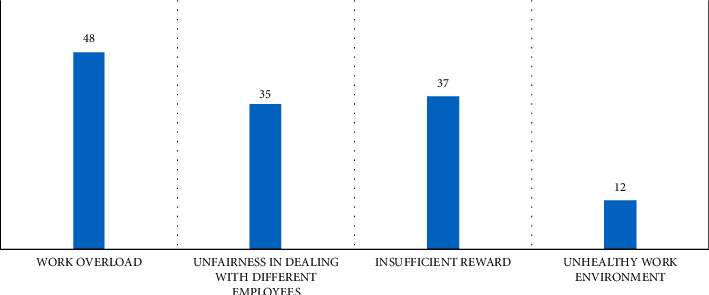
Factors identified by ICU staff that cause occupational burnout.

As shown in Figure 1, respondents connect workload to occupational burnout, while 37 of the respondents believe insufficient workplace rewards increase burnout among ICU staff. About 35 participants noted that unfairness in dealing with employees is a source of burnout. Moreover, 12 subjects stated the workplace environment is a source of burnout for ICU staff.

**Figure 2:**
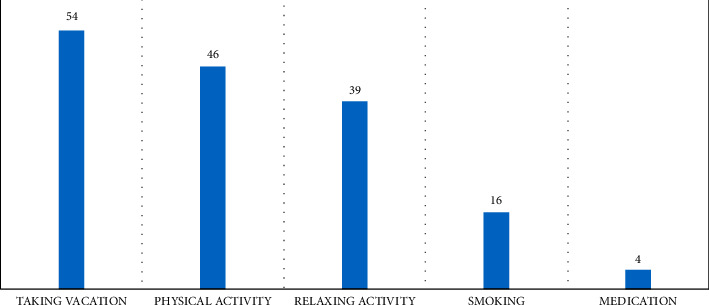
Factors identified by ICU staff that help cope with occupational burnout.

Figure 2 shows the factors identified by ICU staff as occupational burnout coping mechanisms. In this study, 54 participants attested that taking a break and going for a vacation are the best coping mechanisms for occupational burnout. Physical activity comes in second place in reducing occupational burnout among ICU staff, with 46 of the participants noting that it is vital to engage in physical activity to reduce burnout. Relaxing activities and smoking were identified by 39 and 16 subjects, respectively, as coping mechanisms. A few participants (four) identified medication as a way to manage occupational burnout.

## 4. Discussion

ICU staff are exposed to much pressure from varying stressors in their environment. The results show that occupational burnout among ICU nurses, physicians, and respiratory therapists is significant. The indicators of emotional exhaustion, depersonalization, and personal accomplishment are at higher levels. In the results, it is evident in the three subscales that the score is higher compared to the MBI norm sample. A high score indicates that the ICU staff had higher burnout compared to the sample population.

Good management of workplace burnout should be in place to ensure that nurses, respiratory therapists, and physicians can manage workplace stress. In this context, it will be essential to define the level of severity; the staff are exposed to while offering their services in the ICU. On the emotional exhaustion, depersonalization, and personal accomplishment subscales, a high burnout level was indicated in 34%, 27%, and 45% of ICU staff, respectively. A previous study from Saudi Arabia indicated that 54.3%, 46.4%, and 24.3% of the ICU staff had high burnout level in emotional exhaustion, depersonalization, and personal accomplishment subscales [12]. Also, a study in the UK reported that for emotional exhaustion, depersonalization, and personal accomplishment, ICU staff in the high-risk bracket represent 38%, 34%, and 37%, respectively [27].

The group at the highest risk of emotional exhaustion among ICU staff was respiratory therapists (50%), followed by nurses (with 37%) and, finally, physicians with 23%. The cause of burnout, indicated by emotional exhaustion, is categorical to the nature of the tasks that one is undertaking [3]. Burnout is defined by the nature of the activities staff members complete as they make the nature of the impact evident in the process. It is essential to have the necessary controls in place to reduce occupational burnout in the long run.

Depersonalization was the highest among nurses (43.5%), followed by respiratory therapists (33.3%) and physicians (24%). The high risk of developing depersonalization is directly connected to the amount of occupational burnout that the groups record in their respective areas of work.

The leading cause of burnout among ICU staff in the study was workload. Understanding how workers are affected by the workload renders them incapable of resilience in situations. Insufficient workplace rewards and unfairness in dealing with employees were also identified as sources of burnout [21].

Different factors define the ability of ICU staff to cope with occupational burnout in the workplace. Most ICU workers in this study identified taking vacations as the most efficient way of managing workplace burnout. Xie et al. [28] revealed that mindfulness is an important intervention, just like taking a vacation, to solve burnout issues among ICU workers. Taking a break and going for a vacation brings new energy to staff when they return to work [10]. Physical activity was the second most cited factor in reducing occupational burnout among ICU staff. The goal is to create a break in the work environment to rejuvenate [14]. In getting relief from burnout, 39 ICU staff members perceived relaxing activities as a good starting point in this context. Relaxing activities help escape the regular ICU work schedule, hence helping workers relax. Another 16 participants indicated that smoking is an excellent way to reduce workplace burnout. It is a negative reward that nurses, physicians, and respiratory therapists perceive as a perfect way to relieve workplace stress [10].

Interesting is that physical activity and mindfulness training may be used to address burnout. In general, spirituality is useful to address burnout and negative consequences of the pandemic [29, 30]. However, these interventions should be placed in the framework of occupational health surveillance or workplace health promotions programs [31]. Chirico et al. [32] addressed the strategy to tackle challenges in the workplace, especially during the COVID-19 pandemic.

The strength of this study is the use of MBI tool, which is the most used tool and allows to carry out a meta-analysis and evidence-based literature on burnout [33]. However, this study was conducted during the COVID-19 pandemic, and the results could be different if conducted before or after the pandemic.

## 5. Conclusion

Policymakers should be willing to carry out the necessary regulations to ensure that employees are adequately having a low workload. The primary indicators of occupational burnout, emotional exhaustion, depersonalization, and personal accomplishment, were exhibited at varying risk levels by nurses, physicians, and respiratory therapists. The best course to reduce occupational burnout is taking a vacation to stay away from the workplace. Workplace burnout is much more severe among nurses, as they have more prolonged exposure to patients than the other two groups. The reduction of workload helps reduce the burden of the tasks to be accomplished in a working session by the ICU staff. Understanding the course of action in occupational burnout management will make it easy to reduce its impact on ICU workers.

## Figures and Tables

**Table 1:** Participant characteristics and mean (SD) of the MBI subscale scores of ICU staff according to sociodemographic, environmental, and work variables.

Mean (SD) of the MBI subscale score				
Characteristic	*N* = 104 (*n* (%))	Emotional exhaustion	Depersonalization	Personal accomplishment
Gender				
Female	63 (61%)	**22.83 (16.44)**	**9.84 (8.27)**	**29.13 (13.09)**
Male	41 (39%)	**21.85 (12.41)**	**8.17 (5.89)**	**30.27 (11.75)**
Age				
18–24 years old	5 (4.8%)	**35.6 (16.07)**	**15.8 (8.44)**	**29.6 (12.14)**
25–34 years old	60 (58%)	20.75 (15.1)	**8.63 (7.39)**	**29.78 (13.36)**
35–44 years old	33 (32%)	**24.09 (14.6)**	**9.48 (7.31)**	**29.76 (11.58)**
45 and above	6 (5.8%)	19.33 (9.44)	7.5 (6.72)	**26.5 (11.76)**
Education				
Diploma	16 (15%)	18.69 (13.6)	9.13 (6.41)	**25.94 (13.97)**
Bachelor's degree	72 (69%)	**22.83 (15.92)**	9.1 (7.94)	**30.56 (12.51)**
Master's degree	8 (7.7%)	**28.38 (13.56)**	**10.63 (5.97)**	**31.5 (7.46)**
Doctorate degree	8 (7.7%)	20.5 (6.97)	**8.63 (6.86)**	**26.13 (13.82)**
Occupation				
Nurse	62 (60%)	**23.4 (16.21)**	**9.98 (8.15)**	**29.87 (13.15)**
Physician	30 (29%)	20.03 (12.02)	7.33 (5.81)	**27.03 (12.15)**
Respiratory therapist	12 (12%)	**23.5 (15.04)**	**9.67 (6.91)**	**34.42 (8.83)**
Years of experience				
0–5 years	40 (38%)	**24.45 (14.71)**	**10.4 (8.02)**	**31.13 (13.05)**
6–10 years	34 (33%)	20.03 (15.31)	7.24 (6.06)	**27.53 (12.76)**
>10 years	30 (29%)	**22.5 (14.87)**	**9.77 (7.82)**	**29.83 (11.61)**
Health status perception				
Good	87 (84%)	20.53 (14.68)	8.53 (7.52)	**29.56 (13.2)**
Fair	10 (9.6%)	**31.6 (13.01)**	**11.4 (7.49)**	**31 (8.59)**
Poor	7 (6.7%)	**33.14 (12.46)** ^*∗*^	**14.14 (3.39)** ^*∗∗*^	**27.71 (8.94)**
Satisfaction with work				
Good	65 (62%)	18.88 (14.75)	**8.23 (7.85)**	**29.35 (13.18)**
Intermediate	29 (28%)	**25.59 (11.94)**	**9.59 (6.33)**	**28.1 (12.13)**
Poor	10 (9.6%)	**36.5 (14.65)** ^*∗∗*^	**14.2 (5.88)** ^*∗*^	35.3 (7.75)
Type of hospital				
Public	82 (79%)	**23.83 (15.5)**	**9.26 (7.61)**	**30.21 (12.3)**
Private	22 (21%)	17.27 (11.4)^*∗*^	**8.91 (6.9)**	**27.23 (13.37)**
Weekly over time				
0–5 hours	33 (32%)	15.73 (11.73)	6.82 (6.91)	**27.67 (13.67)**
6–10 hours	32 (31%)	**26.44 (14.28)**	**11.28 (7.43)**	**31.47 (11.64)**
>10 hours	39 (38%)	**24.85 (16.21)** ^*∗∗*^	**9.46 (7.48)** ^*∗*^	**29.64 (12.31)**
Shift duty				
Alternant	76 (73%)	20.09 (14.54)	8.09 (7.1)	**28.38 (13.17)**
Day	25 (24%)	**28 (14.61)**	**11.8 (7.84)**	**32.16 (10.49)**
Night	3 (2.9%)	**35.67 (11.06)**	**15 (6.24)**	38.33 (2.08)^*∗∗∗*^
Marital status				
Single	34 (33%)	**23.06 (14.67)**	**9.5 (8.22)**	**31 (13.72)**
Married	68 (65%)	**22.12 (15.36)**	**9.04 (7.16)**	**28.81 (11.98)**
Divorced	2 (1.9%)	**23 (4.24)**	**8.5 (4.95)**	**31.5 (14.85)**
Household income (SAR monthly)				
5000–10000	76 (73%)	**22.95 (15.77)**	**9.59 (7.72)**	**30.61 (12.71)**
11000–20000	16 (15%)	18.38 (13.17)	7.13 (6.93)	**25.69 (11.38)**
21000–30000	6 (5.8%)	**31.67 (9.09)**	**9.83 (5.08)**	**32.83 (8.42)**
>31000–40000	3 (2.9%)	**20 (9.54)**	**14 (7.81)**	**28 (18.03)**
40000	3 (2.9%)	15.33 (7.57)	3.67 (2.89)	**19.33 (14.01)**

*n,* number of individuals; SD, standard deviation. ^*∗*^*P* < 0.05; ^*∗∗*^*P* < 0.01; ^*∗∗∗*^*P* < 0.01; blanks in the table indicate an insignificant *P* value for the three subscales in that test. The mean of the MBI subscale scores that are higher than the MBI norm sample is highlighted in bold.

**Table 2:** Means and standard deviations for the subscale scores of ICU staff compared to the overall sample of Maslach burnout inventory norms.

Subscale	ICU staff (*N* = 104)		MBI norms sample (*N* = 11067)	
	Mean	SD	Mean	SD
Emotional exhaustion	22.44	14.92	21.0	10.8
Depersonalization	9.18	7.44	8.7	5.9
Personal accomplishment	29.58	12.53	34.6	7.1

**Table 3:** Interpretation of MBI subscale scores for ICU staff (*N* = 104).

	High burnout level *n* (%)	Moderate burnout level *n* (%)	Low burnout level *n* (%)
Emotional exhaustion			
Physician	7 (23)	9 (30)	14 (47)
Respiratory therapist	6 (50)	2 (16.7)	4 (33.3)
Nurse	23 (37)	12 (19)	27 (43.5)
Total	36 (34)	23 (22)	45 (43)
Depersonalization			
Physician	7 (24)	8 (27.6)	14 (48)
Respiratory therapist	4 (33.3)	4 (33.3)	4 (33.3)
Nurse	27 (43.5)	9 (14.5)	26 (42)
Total	28 (27)	29 (28)	47 (45)
Personal accomplishment			
Physician	16 (53)	9 (30)	5 (17)
Respiratory therapist	4 (6)	2 (3)	64 (91)
Nurse	17 (27)	17 (27)	28 (45)
Total	47 (45)	20 (19)	37 (35.6)

## Data Availability

The data used to support the findings of this study are available from the corresponding author upon request.
